# Tolvaptan: A Novel Diuretic in Heart Failure Management

**Published:** 2016-01-13

**Authors:** Hilman Zulkifli Amin, Siska Suridanda Danny

**Affiliations:** 1 *Faculty of Medicine, Universitas Indonesia, Jakarta, Indonesia.*; 2 *Department of Cardiology and Vascular Medicine, Faculty of Medicine, Universitas Indonesia, Jakarta, Indonesia.*

**Keywords:** *Heart failure*, *Arginine vasopressin*, *Tolvaptan*, *Drug therapy*

## Abstract

Heart failure (HF) is still a major problem worldwide with high morbidity and mortality rates. The recently developed medication for HF is still incapable of reducing its morbidity and mortality, and clinical data supporting the efficacy and safety of its mainstay therapy remain insufficient.

Arginine-vasopressin (AVP) plays important roles in circulatory and water homeostasis, one of which is water retention through the V_2 _receptor. In patients with HF, there is an increased level of AVP, contributing to such symptoms as edema, dyspnea, and congestion. Tolvaptan as a selective V_2 _receptor antagonist, in addition to the conventional therapy, has been shown to cause an increase in net fluid loss, a decrease in body weight, and a reduction in the rate of HF exacerbation. Such evidence has been provided by the Acute and Chronic Therapeutic Impact of a Vasopressin Antagonist (ACTIV) in Congestive Heart Failure (CHF), Efficacy of Vasopressin Antagonism in Heart Failure Outcome Study With Tolvaptan (EVEREST), Acute Heart Failure Volume Control Multicenter Randomized (AVCMA), and Study of Ascending Levels of Tolvaptan in hyponatremia 1and 2 (SALT-1 and SALT-2) trials. Tolvaptan can be an alternative diuretic in conjunction with other standard therapies for HF and has already been proved to be able to decrease morbidity and mortality, especially in HF patients with hyponatremia.

## Introduction

Heart failure (HF) is a growing global problem, with over 23 million people around the world affected.^1^ The prevalence of HF follows an exponential pattern, and it rises with age.^2^ This disease carries substantial risk of morbidity and mortality. Over 2.4 million patients are hospitalized, and nearly 300 000 deaths annually are directly attributable to HF.^1^

Despite advanced medical care for patients with HF, the average 5-year survival is about 50%.^3^ Currently, various types of therapeutic agents are used for HF as the standard treatment - including diuretics, angiotensin-receptor blockers (ARB), angiotensin-converting enzymes inhibitors (ACE-I), and beta-blockers.^4^ These drugs still play an important role in the treatment of HF patients. Based on the 2012 guideline of the European Society of Cardiology for HF management, standard therapy for HF management is the initiation of combination diuretics and ACE-I or ARB. Subsequently, beta-blockers and other supportive drugs and devices can be added to manage the patient’s clinical condition.^5^

Diuretics are usually the first-line therapy as the fluid removal is an important component of HF treatment to improve oxygenation and relieve the signs and symptoms of edema.^6^ The benefits of diuretics in terms of mortality and morbidity in patients with HF have yet to be clearly elucidated - unlike those of ACE-I and beta-blockers, which are already known as drugs with class of recommendation I and A level of evidence. 

The Cooperative North Scandinavian Enalapril Survival Study (CONSENSUS) and Studies of Left Ventricular Dysfunction (SOLVD)- Treatment respectively revealed that ACE-I can reduce mortality in patients with HF with relative risk reduction (RRR) of 27% and 16%.^5^ The SOLVD study also reported reduced rates of hospitalization with RRR of 26%. Three randomized clinical trials reported that beta-blockers showed the same benefit. The Carvedilol Prospective Randomized Cumulative Survival (Copernicus), Metoprolol CR/XL Randomized Intervention Trial in Congestive Heart Failure (MERIT-HF), and Cardiac Insufficiency Bisoprolol Study (CIBIS) II revealed reduced mortality (each study showed RRR~34%) and HF hospitalization (RRR~28-36%) within about 1 year of starting treatment. ACE-I has been shown to be effective in left ventricular remodeling, whereas beta-blockers have a role in ejection fraction improvement.^5^ Unfortunately, they are not enough to reduce mortality and have created relatively no significant changes over the past several decades. However, other drugs in tandem with the standard therapy can be an alternative therapy and decrease morbidity and mortality in patients suffering from HF - especially those with hyponatremia state, which is commonly found in clinical settings.

Although diuretics have exhibited benefits only in terms of relieving signs and symptoms of HF, they are still important for the improvement of quality of life in patients with HF. Nonetheless, they might worsen the renal function and prognosis of such patients.^7^ In addition, clinical data supporting the efficacy and safety of diuretic therapy remain insufficient. Therefore, improvement in HF treatment requires the discovery of novel agents.^3^ We herein review the possible role for vasopressin antagonists as novel diuretic agents in the management of HF.

***Arginine-Vasopressin Regulation***

**Figure 1:**
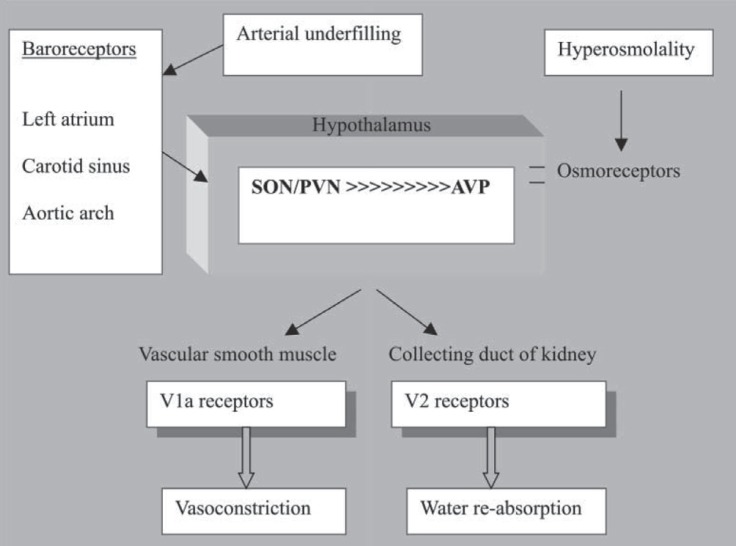
Regulation of water balance by arginine-vasopressin (AVP). Osmoreceptors residing in the anteroventral third ventricle region of the hypothalamus detect decreases in serum osmolality, thereby stimulating the production of AVP. Baroreceptors located in the left atrium, carotid sinus, and aortic arch detect arterial underfilling and stimulate neurons in the supraoptic nucleus (SON) and paraventricular nucleus (PVN) to produce AVP. (The atrial receptors are mediated by the vagus nerve rather than blood pressure.) The neurons of the SON and PVN project into the posterior pituitary gland, where AVP is initially stored and then released into the circulation. V_1a_ receptors, located in the vascular smooth muscle, sense increased levels of AVP and cause vasoconstriction. AVP also stimulates V_2_ receptors, located in the collecting duct of the kidney, causing free water absorption. (Adapted from Sanghi P, Uretsky BF, Schwarz ER. Vasopressin antagonism: a future treatment option in heart failure. Eur Heart J. 2005; 26:538-43, by permission of Oxford University Press.)^12^

The main role of arginine-vasopressin (AVP), or antidiuretic hormone, is to control the body water’s content and blood pressure by affecting the rate of water excretion through the kidney.^8^ AVP is secreted from the posterior pituitary in response to elevation in plasma osmolality and decreases in arterial pressure.^9^ Osmotic pressure is the most sensitive stimulus for AVP release and is mediated by osmoreceptors in the hypothalamus.^10^ Sodium concentration greatly influences osmotic pressure. Baroreceptors - located in the carotid artery, aortic arch, and left atrium - respond to the reduced blood pressure and stimulate neurons located in the supraoptic nuclei and paraventricular nuclei of the hypothalamus to make vasopressin.^11^^, ^^12^ The scheme for a summary of water balance regulation is depicted in Figure 1. 

**Figure 2:**
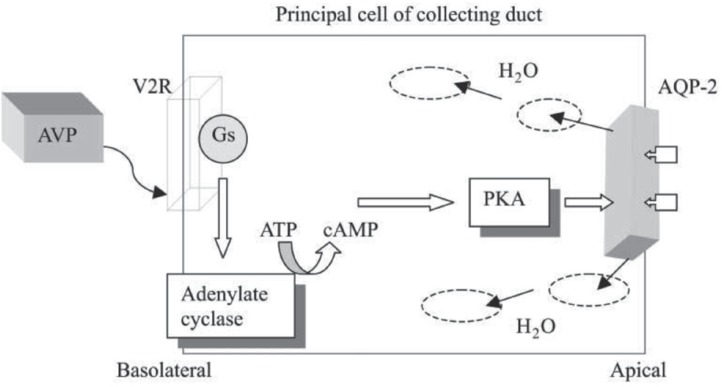
Arginine-vasopressin (AVP) signal transduction pathway in the collecting duct. Vasopressin binds to the V_2_ receptor on the basolateral surface of the principal cells of the collecting duct of the kidney. The receptor couples to Gs (a heterotrimeric GTP-binding protein), which then binds to adenylate cyclase and increases cyclic adenosine monophosphate (cAMP) production. Protein kinase A (PKA) is a multimeric protein. When activated by cAMP, it phosphorylates the acuaporin-2 (AQP-2) molecule, where it is delivered via cytoplasmic vesicles to the apical surface of the collecting duct. The water channels then allow a single file of water molecules to traverse the apical membrane in response to the osmotic gradient where they are returned to the circulation. (Adapted from Sanghi P, Uretsky BF, Schwarz ER. Vasopressin antagonism: a future treatment option in heart failure.Eur Heart J. 2005; 26:538-43, by permission of Oxford University Press.)^12^

ATP, Adenosine triphosphate; V2R, Vasopressin 2 receptor

There are 3 types of AVP receptors in the human body: V_1a_, V_1b_, and V_2 _receptors.^11^ V_1a_ receptors are located at vascular smooth muscle cells (in the myocardium), hepatocytes, and platelets. V_1b_ receptors are found in the anterior pituitary, and V_2_ receptors are located on the basolateral membrane of the kidney collecting tubules and have roles associated with free water absorption by activating the aquaporin (AQP)-2 channel on the apical plasma membrane of the collecting duct cells.^13^ The binding of AVP to V_2_ receptors activates adenylate cyclase, which leads to an increase in intracellular cyclic adenosine monophosphate (cAMP).^14^ This cAMP elevation causes a possible translocation of AQP-2 channels from the intracellular vesicles to the apical plasma membrane through the activation of protein kinase, resulting in a rise in water permeability.^13^ The AVP signal transduction pathway in the collecting duct is depicted in Figure 2.

A previous study reported elevated levels of AVP in patients with HF.^15^ Such elevated levels of AVP result in the impaired excretion of free water in patients suffering from HF due to an increase in the number of AQP-2 channels in the collecting duct, causing water retention and congestion.^13^^, ^^16^

***Studies on Vasopressin-Receptor Antagonist (Tolvaptan)***

Tolvaptan is an orally active, non-peptide, selective V_2_ receptor antagonist.^17^ Selective AVP V_2_ receptor antagonists induce hypotonic diuresis without significantly influencing the excretion of electrolytes.^18^ Ensuing oral administration of tolvaptan at a minimum 40% of an oral dose is absorbed and is highly protein bound (99%). Additionally, it attains top concentrations in 2 to 4 hours without any effects from food, with 12 hours of half-life.^17^

Tolvaptan has been evaluated by many studies. The Acute and Chronic Therapeutic Impact of a Vasopressin Antagonist in Congestive Heart Failure (ACTIV in CHF) is a randomized and double-blinded trial conducted at 45 centers in the United States and Argentina with 319 patients enrolled to study the effects of tolvaptan in hospitalized patients with HF. In addition to the standard therapy, tolvaptan was administered to patients with the signs and symptoms of congestive HF and left ventricular ejection fraction < 40%. Compared with the standard therapy group, the treatment group showed an increase in net fluid loss, a decrease in body weight, and a reduction in the rate of HF exacerbation. Also, there were no adverse effects such as changes in blood pressure, heart rate, or electrolytes.^19^

The Efficacy of Vasopressin Antagonism in Heart Failure Outcome Study with Tolvaptan (EVEREST) trials were 2 multicenter, randomized, double-blind, placebo-controlled studies that also assessed the effects of tolvaptan in hospitalized patients with HF. A total of 4133 patients were admitted and randomized to receive tolvaptan (30 mg/d) or placebo, in addition to the standard HF therapy, within 48 hours of admittance. There was an improvement in body weight, peripheral edema, and patient-assessed dyspnea compared to the control group. However, there was no distinction in the primary end points of all-cause mortality, composite of cardiovascular death and HF hospitalization, or overall quality of life scores between the 2 groups.^20^^-^^22^ Based on this evidence, tolvaptan could not be an inceptive standard therapy.

The Acute Heart Failure Volume Control Multicenter Randomized (AVCMA) trial studied 109 patients with acute decompensated HF and randomly classified them to tolvaptan or carperitide treatment groups. The results showed that urine volume was remarkably higher in the tolvaptan group with fewer unfavorable events such as aggravating HF and hypotension. Nevertheless, subjective symptoms and plasma brain natriuretic peptide (BNP) were similarly improved in both groups.^23^

The Study of Ascending Levels of Tolvaptan in hyponatremia 1 and 2 (SALT-1 and SALT-2) showed that tolvaptan is an efficacious and safe therapy - especially for the treatment of hyponatremic patients with various etiologies such as HF, liver cirrhosis, and syndrome of inappropriate antidiuretic hormone secretion (SIADH).^24^^, ^^25^ Tolvaptan usage is associated with a shorter hospital length of stay than is placebo among patients with SIADH.^24^

***Tolvaptan Profile***

Tolvaptan is designated for patients with clinically eloquent euvolemic or hypervolemic hyponatremia defined as Na^+^ (< 125 mEq/L) and also designated for mild hyponatremia (Na^+^ < 125-135 mEq/L) in symptomatic HF. Other diseases for which tolvaptan can be prescribed are cirrhosis and SIADH.^17^ The initial use of tolvaptan should be monitored in a hospital setting, not only to observe therapeutic responses, but also to prevent the rapid correction of hyponatremia. The initial dose is usually 15 mg/d without regard to meals - with dose adjustments after at least 24 hours, up to a limit of 60 mg/d. Dosage adjustment is not needed regarding age, gender, and race as well as cardiac, renal, and hepatic function. One study demonstrated that no benefit could be gained in anuric patients.^17^

Common unfavorable effects of tolvaptan include thirst, dry mouth, asthenia, constipation, and pollakiuria.^17^ Tolvaptan is metabolized via cytochrome P450 3A4 (CYP3A4).^26^ Other CYP3A4 drug inhibitors like ketoconazole, cyclosporine, clarithromycin, amiodarone, fluoxetine, cimetidine, and midazolam may increase the plasma concentrations of tolvaptan. Nonetheless, CYP3A4 drug inducers like rifampin may decrease the plasma concentrations of tolvaptan by 85%. 

## Conclusion

AVP causes water retention through the V_2 _receptor to maintain the blood pressure. In patients with HF, there is an increased level of AVP, contributing to such symptoms as edema, dyspnea, and congestion. Despite the conventional therapy, tolvaptan as a selective V_2 _receptor antagonist could be a new hope for HF treatment. Trials such as the ACTIV in CHF, EVEREST, AVCMA, and SALT-1, and SALT-2 have demonstrated that tolvaptan - in addition to the initial standard conventional therapy for HF - successfully improves net fluid loss, body weight decrease, and rate of worsening HF. Also, there have been no reports of any serious adverse effects like hypotension and renal function exacerbation. However, no distinction has been reported in the primary end points of all-cause mortality, composite of cardiovascular death and HF hospitalization, or overall quality of life scores by tolvaptan. Accordingly, tolvaptan cannot be used as the initial standard therapy. Thus, further studies are required to directly compare the effects of the standard therapy and tolvaptan in HF management. Although tolvaptan is still known as a highly priced medication, it could be an alternative diuretic in conjunction with other standard therapies for HF that are known to decrease morbidity and mortality in HF, especially in HF patients with hyponatremia. 

## Notes:

***This paper should be cited as:*** Amin HZ, Danny SS. Tolvaptan: A Novel Diuretic in Heart Failure Management. J Teh Univ Heart Ctr 2016;11(1):1-5.

## References

[1] Bui AL, Horwich TB, Fonarow GC (2011). Epidemiology and risk profile of heart failure. Nat Rev Cardiol.

[2] Joseph SM, Cedars AM, Ewald GA, Geltman EM, Mann DL (2009). Acute decompensated heart failure: contemporary medical management. Tex Heart Inst J.

[3] Croft JB, Giles WH, Pollard RA, Keenan NL, Casper ML, Anda RF (1999). Heart failure survival among older adults in the United States: a poor prognosis for an emerging epidemic in the Medicare population. Arch Intern Med.

[4] Hunt SA, American College of Cardiology (2005). American Heart Association Task Force on Practice Guidelines (Writing Committee to Update the 2001 Guidelines for the Evaluation and Management of Heart Failure) ACC/AHA 2005 guideline update for the diagnosis and management of chronic heart failure in the adult: a report of the American College of Cardiology/American Heart Association Task Force on Practice Guidelines (Writing Committee to Update the 2001 Guidelines for the Evaluation and Management of Heart Failure). J Am Coll Cardiol.

[5] McMurray JJ, Adamopoulos S, Anker SD, Auricchio A, Böhm M, Dickstein K, Falk V, Filippatos G, Fonseca C, Gomez-Sanchez MA, Jaarsma T, Køber L, Lip GY, Maggioni AP, Parkhomenko A, Pieske BM, Popescu BA, Rønnevik PK, Rutten FH, Schwitter J, Seferovic P, Stepinska J, Trindade PT, Voors AA, Zannad F, Zeiher A, Bax JJ, Baumgartner H, Ceconi C, Dean V, Deaton C, Fagard R, Funck-Brentano C, Hasdai D, Hoes A, Kirchhof P, Knuuti J, Kolh P, McDonagh T, Moulin C, Popescu BA, Reiner Z, Sechtem U, Sirnes PA, Tendera M, Torbicki A, Vahanian A, Windecker S, McDonagh T, Sechtem U, Bonet LA, Avraamides P, Ben Lamin HA, Brignole M, Coca A, Cowburn P, Dargie H, Elliott P, Flachskampf FA, Guida GF, Hardman S, Iung B, Merkely B, Mueller C, Nanas JN, Nielsen OW, Orn S, Parissis JT, Ponikowski P, Task Force for the Diagnosis and Treatment of Acute and Chronic Heart Failure 2012 of the European Society of Cardiology, ESC Committee for Practice Guidelines, ESC guidelines for the diagnosis and treatment of acute and chronic heart failure 2012: The Task Force for the Diagnosis and Treatment of Acute and Chronic Heart Failure 2012 of the European Society of Cardiology Developed in collaboration with the Heart Failure Association (HFA) of the ESC (2012). Eur J Heart Fail.

[6] Dohi K, Ito M (2014). Novel diuretic strategies for the treatment of heart failure in Japan. Circ J.

[7] Suzuki S, Yoshihisa A, Yamaki T, Sugimoto K, Kunii H, Nakazato K, Abe Y, Saito T, Ohwada T, Suzuki H, Saitoh S, Kubota I, Takeishi Y, AVCMA investigators (2015). Vasopressin V2 receptor antagonist tolvaptan is effective in heart failure patients with reduced left ventricular systolic function and low blood pressure. Int Heart J.

[8] Ishikawa SE, Schrier RW (2003). Pathophysiological roles of arginine vasopressin and aquaporin-2 in impaired water excretion. Clin Endocrinol (Oxf).

[9] Lee CR, Watkins ML, Patterson JH, Gattis W, O'connor CM, Gheorghiade M, Adams KF Jr (2003). Vasopressin: a new target for the treatment of heart failure. Am Heart J.

[10] Dixon MB, Lien YH (2008). Tolvaptan and its potential in the treatment of hyponatremia. Ther Clin Risk Manag.

[11] Guyton AC, Hall JE, Guyton AC, Hall JE (2006). The body fluid and kidneys. Textbook of Medical Physiology.

[12] Sanghi P, Uretsky BF, Schwarz ER (2005). Vasopressin antagonism: a future treatment option in heart failure. Eur Heart J.

[13] Nielsen S, Kwon TH, Christensen BM, Promeneur D, Frøkiaer J, Marples D (1999). Physiology and pathophysiology of renal aquaporins. J Am Soc Nephrol.

[14] Birnbaumer M (2000). Vasopressin receptors. Trends Endocrinol Metab.

[15] Francis GS, Benedict C, Johnstone DE, Kirlin PC, Nicklas J, Liang CS, Kubo SH, Rudin-Toretsky E, Yusuf S (1990). Comparison of neuroendocrine activation in patients with left ventricular dysfunction with and without congestive heart failure A substudy of the Studies of Left Ventricular Dysfunction (SOLVD). Circulation.

[16] Chin MH, Goldman L (1996). Correlates of major complications or death in patients admitted to the hospital with congestive heart failure. Arch Intern Med.

[17] Otsuka Pharmaceutical Company Samsca® (tolvaptan): highlights of prescribing information.

[18] Izumi Y, Miura K, Iwao H (2014). Therapeutic potential of vasopressin-receptor antagonists in heart failure. J Pharmacol Sci.

[19] Gheorghiade M, Gattis WA, O'Connor CM, Adams KF Jr, Elkayam U, Barbagelata A, Ghali JK, Benza RL, McGrew FA, Klapholz M, Ouyang J, Orlandi C (2004). Acute and Chronic Therapeutic Impact of a Vasopressin Antagonist in Congestive Heart Failure (ACTIV in CHF) Investigators Effects of tolvaptan, a vasopressin antagonist, in patients hospitalized with worsening heart failure: a randomized controlled trial. JAMA.

[20] Gheorghiade M, Orlandi C, Burnett JC, Demets D, Grinfeld L, Maggioni A, Swedberg K, Udelson JE, Zannad F, Zimmer C, Konstam MA (2005). Rationale and design of the multicenter, randomized, double-blind, placebo-controlled study to evaluate the Efficacy of Vasopressin antagonism in Heart Failure: Outcome Study with Tolvaptan (EVEREST). J Card Fail.

[21] Gheorghiade M, Konstam MA, Burnett JC Jr, Grinfeld L, Maggioni AP, Swedberg K, Udelson JE, Zannad F, Cook T, Ouyang J, Zimmer C, Orlandi C (2007). Efficacy of Vasopressin Antagonism in Heart Failure Outcome Study With Tolvaptan (EVEREST) Investigators Short-term clinical effects of tolvaptan, an oral vasopressin antagonist, in patients hospitalized for heart failure: the EVEREST Clinical Status Trials. JAMA.

[22] Blair JE, Zannad F, Konstam MA, Cook T, Traver B, Burnett JC Jr, Grinfeld L, Krasa H, Maggioni AP, Orlandi C, Swedberg K, Udelson JE, Zimmer C, Gheorghiade M, EVEREST Investigators (2008). Continental differences in clinical characteristics, management, and outcomes in patients hospitalized with worsening heart failure results from the EVEREST (Efficacy of Vasopressin Antagonism in Heart Failure: Outcome Study with Tolvaptan) program. J Am Coll Cardiol.

[23] Suzuki S, Yoshihisa A, Yamaki T, Sugimoto K, Kunii H, Nakazato K, Abe Y, Saito T, Ohwada T, Suzuki H, Saitoh S, Kubota I, Takeishi Y, AVCMA investigators (2013). Acute heart failure volume control multicenter randomized (AVCMA) trial: comparison of tolvaptan and carperitide. J Clin Pharmacol.

[24] Dasta JF, Chiong JR, Christian R, Lin J (2012). Evaluation of costs associated with tolvaptan-mediated hospital length of stay reduction among US patients with the syndrome of inappropriate antidiuretic hormone secretion, based on SALT-1 and SALT-2 trials. Hosp Pract (1995).

[25] Cleland JG, Coletta AP, Abdellah AT, Nasir M, Hobson N, Freemantle N, Clark AL (2007). Clinical trials update from the American Heart Association 2006: OAT, SALT 1 and 2, MAGIC, ABCD, PABA-CHF, IMPROVE-CHF, and percutaneous mitral annuloplasty. Eur J Heart Fail.

[26] Yi JH, Shin HJ, Kim HJ (2011). V2 receptor antagonist; tolvaptan. Electrolyte Blood Press.

